# Treatment of Severe Refractory Hematuria due to Radiation-Induced Hemorrhagic Cystitis with Dexamethasone

**DOI:** 10.1155/2017/1560363

**Published:** 2017-06-21

**Authors:** José Carlos Rodrigues Nascimento, Márcio Wilker Soares Campelo, Iuri Arruda Aragão, José Fernando Bastos de Moura, Lúcio Flávio Gonzaga Silva, Reinaldo Barreto Oriá

**Affiliations:** ^1^Laboratory of the Biology of Tissue Healing, Ontogeny and Nutrition, Department of Morphology and Institute of Biomedicine, School of Medicine, Federal University of Ceara, Fortaleza, CE, Brazil; ^2^Unimed Regional Hospital, Fortaleza, CE, Brazil; ^3^Cancer Institute of Ceara, Fortaleza, CE, Brazil; ^4^Department of Clinical Medicine, School of Medicine, Federal University of Ceara, Fortaleza, CE, Brazil

## Abstract

Treatment of pelvic neoplasms with radiotherapy may develop sequelae, especially RHC. An 85-year-old male patient was admitted to a hospital emergency with gross hematuria leading to urinary retention and was diagnosed with RHC. The urinary bladder was probed, unobstructed, and maintained in continuous three-way saline irrigation. During 45 days of hospitalization, the patient underwent two cystoscopic procedures for urinary bladder flocculation, whole blood transfusions, and one platelet apheresis. None of these interventions led to clinical resolution. As the patient hematological condition was deteriorating, dexamethasone (4 mg i.v.,* bolus* of 6/6, 12/12, and 24 h during five days) and epoetin alpha (1000 IU, 1 ml, s.c., for four weeks) were administered which led to the remission of the urinary bleeding. Dexamethasone therapy may be considered for RHC, when conventional treatments are not effective or are not possible, avoiding more aggressive interventions such as cystectomy.

## 1. Introduction

Pelvic neoplasms, such as in the prostate, bladder, rectum, uterus, ovary, and cervix cancer, may be treated with radiotherapy alone or in combination with chemotherapy. Different total dose of radiation varies according to cancer severity and higher doses likely increase the odds of toxicity in the genitourinary system. The urinary bladder is particularly sensitive to low radiation doses and is often more affected than other pelvic tissues [1], especially due to low urothelium cell turnover [2].

RHC accounts for 23% to 80% of all complications related to pelvic radiation, with an incidence of severe hematuria ranging from 5% to 8% [3]. RHC may occur 3 months to even 14 years after radiotherapy and men are more predisposed than women (2.8 : 1), as prostate cancer is often treated with radiotherapy sessions [1, 4]. RHC is characterized by progressive fibrotic obliteration of mucosal arterioles and capillaries, leading to tissue hypoxia and necrosis [3]. Cystoscopy depicts neovascularization and telangiectasia, which can result in severe and refractory macroscopic hematuria [1]. Gross and prolonged hematuria may require successive blood transfusions, several hospital admissions, and surgical treatments to the patient, increasing hospital costs and mortality rates [5, 6].

The diagnosis of hemorrhagic cystitis is defined by anamnesis, urine examination, urinary cytology, and cystoscopy. Computed tomography may be important to rule out upper urinary tract injury as a cause of hematuria and magnetic resonance imaging is important in the presence of prior pelvic tumor diagnosis [3].

Various treatment approaches may be indicated for RHC, such as bladder irrigation; intravesical treatment with alum or formalin and hyaluronic acid; corticoids; oral estrogen, sodium pentosan polysulfate, and hyperbaric oxygen therapy; factor VIIa/factor VIII and epsilon-aminocaproic acid; hydrodistension of the bladder; embolization or ligation of the internal vesical and iliac arteries; and, as a last alternative, cystectomy with urinary diversion [4]. Although an array of treatments is available, RHC is still a challenging condition to the clinician, due to its most often poor treatment efficacy in a short term, especially to more vulnerable individuals, as in the elderly.

## 2. Case Report

An 85-year-old male patient had undergone radical prostatectomy after prostate specific antigen (PSA) rise and in situ prostate cancer diagnosis by rectal examination and biopsy 18 years ago (in 1998). In 2003, radiotherapy sessions were indicated following cancer recurrence suggested by high PSA levels (7.5 ng/mL).

After 13 years of radiotherapy cessation (in 2016), he was admitted to an emergency hospital with urinary retention and macroscopic hematuria. The urinary bladder was probed, unobstructed, and maintained in continuous saline irrigation with three-way catheter and the patient was hospitalized in an in-patient unit. In the admission laboratory tests, renal function and coagulation were within normality (INR: 1.22; platelets: 127,000/*μ*l, urea: 38 mg/dL; creatinine: 0.72 mg/dL; Na^+^: 132 mEq/L and K^+^: 3.4 mEq/L). C-reactive protein was found elevated (6.42 mg/dL) and hemoglobin (Hb) markedly reduced: 7.0 g/dL. Ultrasonography of the abdomen revealed a distended urinary bladder and smooth and regular walls with no amorphous echoes; kidneys showed multiple bilateral cortical cysts and bilateral hydronephrosis. Two emergency cystoscopies were performed for cauterization of the bleeding urinary bladder after prolonged gross hematuria. Radiotherapy-induced hemorrhagic cystitis was clinically diagnosed, without evidence of other pathologies. Urine culture was positive for* Enterococcus faecalis* and treated with ciprofloxacin. After administration of 1 unit of red blood cells (RBC), hemoglobin increased to 8.9 g/dL. In attempt to reduce prolonged hematuria, an arteriography followed by vesical artery catheterization was conducted, but as these vessels were friable, neither vesical nor iliac artery embolization was performed. As the patient clinical condition was deteriorating due to severe hematuria, significant anemia and thrombocytopenia (platelets: 76,000/*μ*L) were found. Three units of RBC in 1 week and 1 platelet apheresis were prescribed. In addition, epoetin alpha (eprex®: 10000 IU, 1 ml, s.c.) was administered weekly to improve the erythropoiesis. Amlodipin was discontinued and replaced with captopril (25 mg daily), as calcium channel blockers may interfere with platelet function [7]. After 25 days of hospitalization, dexamethasone was administered at an attack dose of 4 mg, i.v., bolus of 6/6 hours for three days, 12/12 h for two days, and 24/h for one day, which led to the remission of the urinary bleeding in a gradual manner. The patient completed epoetin alpha treatment and then was discharged after 45 days of hospitalization, without further complaints, and was hemodynamically stable (platelets: 117,00 *μ*l, Hb: 11.7 g/dL). Renal function and coagulation were within normality. C-reactive protein was still high (8.7 mg/dL). The patient was referred for hyperbaric oxygen therapy sessions to fully improve hematuria episodes.

## 3. Discussion

Corticosteroids have not been widely used in the treatment of RHC [8]. Yanagi et al. [9] reported successful treatment of radiation-induced severe hemorrhagic cystitis with oral administration of prednisolone and resolved macroscopic hematuria within 2 weeks. In addition, dexamethasone has been beneficial in models of ifosfamide-induced hemorrhagic cystitis in rats [10].

In this case report, the remission of hematuria was achieved only following a* bolus* treatment of dexamethasone (4 mg) given to the patient; a similar outcome was described by Jin [11] with methylprednisolone (1 g/d) for 5 days. Glucocorticoids have been shown to improve ifosfamide-induced hemorrhagic cystitis, by reducing interleukin-1, tumor necrosis factor alpha cytokines, platelet-activating factor, and inducible nitric oxide synthase activation [12]. Furthermore, corticosteroids may be beneficial to hemorrhagic cystitis by improving hematological parameters. Dexamethasone has been found to promote erythropoiesis by increasing the absolute number of erythroid cells produced from CD34(+) cells and by selectively increasing the number of burst-forming units-erythroid (BFU-E), in addition to reducing TNF-*α* negative effects on erythropoiesis [13]. The combination of dexamethasone and epoetin alpha administered to the patient may have had an important synergic effect in improving anemia and reducing the urinary bladder inflammatory response that could have otherwise favor hemorrhage and anemia.

Endovenously administered dexamethasone was the major satisfactory therapy in improving RHC, which was refractory to cystoscopic flocculation and was an alternative to the vesical artery embolization failure. Caution is needed for applying dexamethasone therapy to vulnerable patients due to its well-known cardiovascular potential side effects. Despite the side effects, the treatment of RHC with dexamethasone should be considered before more radical interventions such as cystectomy. Following hospital discharge, the patient was referred to weekly hyperbaric oxygen therapy sessions. Oral dexamethasone (4 mg) was prescribed to control recurrence of urinary hemorrhagic episodes.

## Abbreviations

RHC: Radiotherapy-induced refractory hemorrhagic cystitis.

## References

[1] Sutani S., Ohashi T., Sakayori M. (2015). Comparison of genitourinary and gastrointestinal toxicity among four radiotherapy modalities for prostate cancer: conventional radiotherapy, intensity-modulated radiotherapy, and permanent iodine-125 implantation with or without external beam radiotherapy. *Radiotherapy and Oncology*.

[2] Zwaans B. M., Nicolai H. G., Chancellor M. B., Lamb L. E. (2016). Challenges and opportunities in radiation-induced hemorrhagic cystitis. *Reviews Urology*.

[3] Browne C., Davis N. F., Mac Craith E. (2015). A narrative review on the pathophysiology and management for radiation cystitis. *Advances in Urology*.

[4] Oliai C., Fisher B., Jani A. (2012). Hyperbaric oxygen therapy for radiation-induced cystitis and proctitis. *International Journal of Radiation Oncology, Biology, Physics*.

[5] Martinez D. R., Ercole C. E., Lopez J. G., Parker J., Hall M. K. (2015). A novel approach for the treatment of radiation-induced hemorrhagic cystitis with the GreenLight™ XPS laser. *International Braz J Urol*.

[6] Bevers R. F. M., Bakker D. J., Kurth K. H. (1995). Hyperbaric oxygen treatment for haemorrhagic radiation cystitis. *The Lancet*.

[7] Folts J. D. (1997). Inhibition of platelet activity in vivo by amlodipine alone and combined with aspirin. *International Journal of Cardiology*.

[8] Liem X., Saad F., Delouya G. (2015). A practical approach to the management of radiation-induced hemorrhagic cystitis. *Drugs*.

[9] Yanagi M., Nishimura T., Kurita S., Lee C., Kondo Y., Yamazaki K. (2011). A case of prednisolone therapy for radiation-induced hemorrhagic cystitis. *The Japanese Journal of Urology*.

[10] Vieira M. M., Castro Brito G. A., Belarmino-Filho J. N. (2003). Use of dexamethasone with mesna for the prevention of ifosfamide-induced hemorrhagic cystitis. *International Journal of Urology*.

[11] Jin J. G. (2014). High-dose methylprednisolone is effective in treating radiation-induced refractory haemorrhagic cystitis. *Internal Medicine Journal*.

[12] Ribeiro R. A., Freitas H. C., Campos M. C. (2002). Tumor necrosis factor-alpha and interleukin-1beta mediate the production of nitric oxide involved in the pathogenesis of ifosfamide induced hemorrhagic cystitis in mice. *The Journal of Urology*.

[13] Narla A., Dutt S., McAuley J. R. (2011). Dexamethasone and lenalidomide have distinct functional effects on erythropoiesis. *Blood*.

